# Dr. Koch’s Official Report of the Cholera in Egypt

**Published:** 1884-02

**Authors:** 


### DR. KOCH'S OFFICIAL REPORT ON THE CHOLERA IN EGYPT, WITH THE RESULTS OF HIS INVESTIGATIONS AND DISCOVERIES, AS CHIEF OF THE GERMAN SCIENTIFIC EXPEDITION.*

As the cholera epidemic was already rapidly on the decline when the Commission arrived in Egypt, it was not to be expected from the very commencement that that country would afford full scope for the investigation. Moreover, as in an epidemic the period of its decline is the least adapted for its ætiological investigation, the original plan was to make the necessary preparatory studies in Egypt, in order, on the epidemic spreading to Syria, to avail ourselves of these in those places, which from having just been invaded by cholora, would have afforded a favorable ground for investigation.

The first part of this plan we have been able so far to carry out according to the wishes of all, for the Commission has found plenty of opportunity, during its stay in Alexandria, of collecting the necessary materials for its preparatory studies. For this success I am chiefly indebted to the kindness of the doctors of

---

* Translated by the *Standard* correspondent fr^m the *Imperial German Gazette.*

the Greek Hospital, who advanced the objects of the expedition in the most effective manner, by placing at our disposal sufficient rooms, as well as the cholera patients who came to the Hospital, and the corpses of those who died from the disease.

At first the Commission occupied for their work two bright rooms adjacent to one another on the ground floor of the hospital. One of them was devoted to the microscopic work, and the other to the inoculation experiments. The animals on which the experiments were made were kept in both rooms. But as their number increased, and as it appeared dangerous to be handling the inoculation-matter in the same rooms in which one spent almost the whole day, the animals for experiments were brought into a room of the old Hospital, distant from the others, where tne experiments of inoculation were made.

The materials we have had up till now for investigation have been twelve cholera patients and twelve cholera corpses. The symptoms corresponded in each case in every detail to those of genuine Asiatic cholera. Small portions of the blood of these patients, of their ejecta and excreta, were taken and examined. As it was very soon evident that the blood was quite free from micro-organisms, and that the ejecta too contained only comparatively few of them, but that the excreta contained a very significant amount of micro-organisms, these were mostly used for the inoculation experiments on the animals. Amongst the dissected subjects the most different nations are represented (three Nubians, two German-Austrians, four Greeks, one Turk), various ages (two children, two adults over sixty years of age, the rest between twenty and thirty-five), and cases of different durations of illness. The most important point, however, is that the bodies could generally be dissected immediately after death, or only a few hours afterwards. The changes which are caused in the organs, and especially early in the intestines, by decomposition, and which render the microscopic investigation of these organs extremely difficult, were thus with certainty avoided. I am inclined to give the more weight to this circumstance, as it will scarcely be possible to obtain in other places subjects so suitable for microscopic investigation. The state of the bodies, as also the symptoms of the disease, left no doubt that we had to dea here with Asiatic cholera, and not—as was asserted at first on many sides—with diseases similar to cholera, so-called choleriform and choleroid complaints. No organized infectious matter could be traced in the blood, or in those organs which in other infectious diseases are generally the seat of micro-parasites—as, for instance in the lungs, the spleen, the kidneys, or the liver. Sometimes bacteria were found in the lungs, which, however, as was evident from their shape and position, had nothing to do with the course of the disease, but had reached the lungs by the inhaling of the ejected contents of the stomach. Micro-organisms in great abundance and of most different kinds were found in the contents of the intestines, and in the excreta of the cholera patients. No one kind was present in great predominance over the others. Special signs were also wanting which could have been attributed to a connection with the process of the disease. On the other hand, the intestines themselves gave an important result. With the exception of one case, which some weeks after getting over the cholera terminated fatally from another complaint, a certain kind of bacteria was found in the coatings of the intestines., These bacteria are stabiform, and belong, therefore, to the bacilli they resemble in size and form the bacilli found in glanders. In those cases in which the intestines by magnifying show the slightest changes the bacilli had penetrated into the utricular glands of the mucous membranes of the intestines, and had caused there a considerable irritation, as the dilation of the opening of the gland, and the collection of granular circular cells in the interior of the gland, showed. In many cases the bacilli had found their way between the epithelia of the gland, and had multiplied between the epithelia and the glandular membrane. The bacilli had also settled in large numbers on the surface of the villi of the intestines, and had often penetrated into their tissue. In severe cases, which terminated in bloody infiltration of the mucous membrane of the intestines, the bacilli were found in very large numbers, and they did not then confine themselves to the invasion of the utricular glands, but passed into the surrounding tissue, into the lower layers of the mucous membrane, and in some places right to the musular skin of the intestine. The intestinal villi were also in such cases penetrated by bacilli.

The chief seat of these changes is in the lower part of the small intestine. If this discovery had not been made in perfectly fresh corpses, one could have made little or no use of it, for the influence of putrefaction is able to bring about similar vegetation of bacteria in the intestines. For this reason I had been unable to attach any value to the fact that I had already, a year ago, found in a cholera-infected intestine, which I had received direct from India, the same bacilli in the same order as now in the Egyptian cholera cases, for I was always obliged to think of a possibility of complication with post-mortem putrefaction. But now this former discovery, which was made in four different Indian cholera subjects, is of considerably greater value, as now the possible error caused by the appearance of putrefaction can be safely set aside. It is also not unimportant that in the similarity between the state of the intestines in the Indian and Egyptian cholera cases a further proof of the identity of both diseases is obtained. The number of cholera subjects that we were able to investigate was certainly small. But as we met with the bacilli in all cases of cholera that were immediately brought under our attention, whilst in the one case that we investigated after the process of cholera was over, and in many other cases of people who died of other diseases, and whom we examined with the same purpose, they were missing, there can be no doubt that they stand in some relation to the operation of cholera. However, from the coincidence of the latter with the finding of bacilli in the mucous membrane of the intestines, we cannot conclude that the bacilli are the causes of cholera. It might be the very reverse, and it could just as well be supposed that the operation of cholera causes such disturbances in the mucous membrane of the intestines, that from the many bacteria that are always parasitic in the intestines, one form of certain bacilli was enabled to penetrate into the tissue of the mucous membrane of the intestine. Which of these assumptions is the correct one—whether the operation of infection, or whether the invasion of bacteria is the primary cause—that can only be decided by trying to isolate the bacteria from the affected tissues, to propagate them artificially, and then by inoculation experiments on animals to reproduce the illness. For this purpose it is absolutely necessary to have such animals at one's disposal as are susceptible to the infectious matter in question. Despite all endeavors, no one has yet indisputably succeeded in making animals ill of cholera.

Several experiments have been made on rabbits, guinea-pigs, dogs, cats, monkeys, pigs, rats, etc., but always without result. The sole statements which in this matter are deserving of attention have been made by Thiersch, who saw a number of mice get diarrhœa, and die after having been fed upon the contents of a cholera-infected intestine. This experiment has been confirmed by trustworthy experimentalists, as for instance Burdon Sanderson, though certainly disputed by others. Anyhow, it was necessary to repeat these experiments, as it is of the greatest importance to find an animal that is susceptible to cholera. For this purpose, fifty mice were brought from Berlin, as it was very improbable that the requisite number of them could be soon procured in Alexandria, and the inoculation experiments were begun on these. Monkeys, too, which are the only species of animal susceptible to some human diseases, such as small-pox, were also used for these experiments. Finally, attempts were made to infect some dogs and poultry. But in spite of all efforts these experiments were without result.

The bacilli found in the contents of the intestines and in the coatings of the intestines were also artificially propagated, and with these, too, experiments were made by giving them as food and partly by vaccination. Some of these produced putrefying illnesses when they were inoculated, but cholera could not be produced from any of them.

That the infectious matter must often be contained in a powerful form in the excreta of cholera patients is proved by much experience, especially by the frequent cases of illness amongst laundresses, who had to wash the linen of the cholera patients. In the Greek Hospital such a case occurred, and a laundress, who was exclusively occupied with the washing of linen from cholera patients, caught cholera.

It is perfectly certain that in the numerous samples made use of, some, at least, contained the infectious matter. If, however, no result was obtained, it may have been because the species of animals used for the experiments were themselves unsusceptible to cholera, or that the correct method of inoculating has not yet been discovered. In both directions the experiments are to be continued and modified, but there is little hope of anything being attained in this direction with the materials at our disposal, for it is not very probable that the reason of the failure of these experiments is to be looked for in these circumstances alone. There is another explanation, for the accuracy of which much can be said. In one of the places visited by the cholera it is known the plague died out long before all the people had taken infection, and although the infectious matter was scattered in great quantities all over the whole place, fewer people became ill, and the epidemic disappeared in the midst of many persons susceptible to the disease. The occurrence can only be explained by the supposition that towards the end of the epidemic the infectious matter loses some of its infective power, or at least is uncertain in its spreading. But if towards the end of the epidemic even human beings are themselves no longer so liable to receive the infectious matter, then one cannot but expect that this also will be the case in experiments with animals, about whose susceptibility to cholera nothing is as yet known. For our experiments we only had at our disposal such objects as had been collected at the end of the epidemic, whose inefficiency was more or less to be pre-supposed. It is possible that under favorable circumstances—for example, at the commencement of the epidemic—the inoculation of animals might succeed, and by this means we might at once get to know if the bacilli traced by me in the mucous membrane of the intestines are the real cause of cholera.

Far as the results hitherto obtained by the Commission are from the solution of the problem, and little as they are adapted for practical use in struggling against cholera, yet, in consideration of the unfavorable circumstances and the short time of investigation, they may be considered good. They entirely answer the original purpose of the preliminary investigation, and in so far exceed this as enough has been effected for the first condition, which has to be fulfilled in inquiring into a contagious disease, by the constant discovery of characteristic micro-organisms, and thereby a fixed boundary has been placed for further investigations.

From the preceding account it can be seen that the Commission has not been able to acquire fresher information for the solution of the problems given to it, than has been obtained hitherto.

Hereupon follows a brief explanation of the localities where the investigations could be continued, concluding thus :

"The only possibility of continuing the investigations is now offered in India, where in several large towns, especially in Bombay, the cholera is prevailing to such an extent that one cannot expect it to die out for some time. There, too, we could without any doubt most easily get attached to a hospital, that having proved of so great advantage in Alexandria."

Acting on the above report, the continuation of the scientific investigations in East India has been allowed, and the Commission will shortly leave for Bombay for this purpose.—*Medical Press.*

